# Complementary function of two transketolase isoforms from *Moniliella megachiliensis* in relation to stress response

**DOI:** 10.1186/s13568-017-0342-0

**Published:** 2017-02-21

**Authors:** Hisashi Iwata, Yosuke Kobayashi, Daiki Mizushima, Taisuke Watanabe, Jun Ogihara, Takafumi Kasumi

**Affiliations:** 1 Applied Microbiology and Biotechnology Laboratory, Department of Chemistry and Lifescience, Nihon University, 1866 Kameino, Fujisawa, Kanagawa 252-0880 Japan; 2 Bioproduction Research Institute, National Institute of Advanced Industrial Science and Technology, 1-1-1 Higashi, Tsukuba, Ibaraki 305-8566 Japan; 3 Biomolecular Engineering Laboratory, National Food Research Institute, National Agriculture and Food Research Organization, 2-1-12, Kannondai, Tsukuba, Ibaraki 305-8542 Japan

**Keywords:** *Moniliella megachiliensis*, Transketolase isogenes, Stress response, Erythritol

## Abstract

Two transketolase isogenes, *MmTKL1* and *MmTKL2,* isolated from *Moniliella megachiliensis* were investigated for their roles in stress response and erythritol biosynthesis. The encoded proteins were highly homologous in amino acid sequence and domain structure. Two stress response elements (STREs) were found upstream of *MmTKL1*, while no STRE was found upstream of *MmTKL2.* In contrast, two Ap-1 elements were present upstream of *MmTKL2*, but none were detected upstream of *MmTKL1*. *MmTKL2* partially complemented the aromatic amino acid auxotrophy of a *Saccharomyces cerevisiae tkl1* deletion mutant, suggesting that at least one of the *MmTKLs* functioned as a transketolase in vivo. In response to short-term osmotic stress (20% glucose or 1.2 M NaCl) in *Moniliella* cells, *MmTKL1* expression increased rapidly through the first 40 min before subsequently decreasing gradually, while *MmTKL2* expression showed no significant change. In contrast, short-term oxidative stress (0.15 mM menadione) induced considerable increases in *MmTKL2*, while *MmTKL1* expression remained low under the same conditions. Long-term osmotic stress (20% glucose) yielded increased expression of both genes starting at 12 h and continuing through 72 h. During either osmotic or oxidative stress, intracellular erythritol accumulation could clearly be correlated with the pattern of expression of either *MmTKL1* or *MmTKL2*. These results strongly suggested that *MmTKL1* is responsible primarily for the response to osmotic stress, while *MmTKL2* is responsible primarily for the response to oxidative stress. Thus, we postulate that the two transketolase isoforms of *M. megachiliensis* play distinct and complementary roles in coordinating erythritol production in response to distinct environmental stresses.

## Introduction

*Moniliella megachiliensis* SN-124A, a yeast-like fungus isolated from dry fruit, is a highly osmotolerant microorganism. This strain grows even in a 60% glucose solution, and produces a significant amount of erythritol [with maximum yields exceeding 40% (w/v)] as an osmoregulatory compatible solute when cultivated in high-glucose medium (de Hoog et al. 2011; Ishizuka et al. 1989). Erythritol, the sugar alcohol of tetrose, is distributed widely in nature, and is present at low levels in fruits, mushrooms, and fermented foods (Shindo et al. 1988; Yoshida et al. 1984). Erythritol is currently used as a low calorie sweetener, having a cool and plain sweetness (70% that of sugar) and low energy value (0.4 kcal/g) due to being non-metabolizable in the human body (Noda et al. 1994). Furthermore, erythritol has been reported to have antioxidant properties: the compound is an efficient hydroxyl radical scavenger and may help protect against hyperglycemia-induced vascular damage (den Hartog et al. 2010). Hence, this polyol could serve as an antioxidant sweetener for use by diabetics. Moreover, erythritol recently has been proposed for use as a feedstock (in place of petrochemicals) for the synthesis of bio-plastics (Amada et al. 2012). Owing to these appealing characteristics, demand for erythritol continues to expand year by year.

In the prokaryotic bacteria *Oenococcus oeni* (formerly *Leuconostoc oenos*), erythritol is synthesized from erythritol-4-phosphate, which is itself generated from fructose-6-phosphate by the action of phosphoketolase (Veiga-da-Cunha et al. 1992, 1993; Richter et al. 2001). In contrast, in eukaryotes like *Moniliella* or *Candida*, erythritol is produced via the pentose phosphate pathway (PPP), whereby the precursor erythrose, formed through dephosphorylation of erythrose-4-phosphate, is enzymatically reduced to erythritol by an NADP^+^-dependent erythrose reductase (ER) (Lee et al. 2003; Ookura et al. 2005; Kim et al. 2013). Previously, we noted that ER, a member of the aldo*–*keto reductase family, is not found in *Saccharomyces cerevisiae*, and that transgenic expression of *ER* (an ER-encoding gene) in *S. cerevisiae* does not provide ER activity, despite the accumulation of ER protein (Kobayashi et al. 2013). Nonetheless, the PPP is considered to play an important role in erythritol biosynthesis (Kobayashi et al. 2013; Sawada et al. 2009). Among the enzymes involved in PPP, transketolase (TKL) is a key enzyme that transfers a ketol group from xylulose-5-phosphate to ribose-5-phosphate, yielding glyceraldehyde-3-phosphate and sedoheptulose-7-phosphate. In addition, TKL can produce fructose-6-phosphate and glyceraldehyde-3-phosphate via ketol transfer from xylulose-5-phosphate to erythrose-4-phosphate (Lindqvist et al. 1992; Schenk et al. 1998; Nikkola et al. 1994). Notably, fructose-6-phosphate and glyceraldehyde-3-phosphate also are intermediates that participate in glycolysis. Hence, TKL is a multi-functional enzyme that modulates both the pentose phosphate and glycolytic pathways in the cell.

*Escherichia coli* harboring a knock-out of the TKL-encoding gene require aromatic amino acids (AAAs) for normal growth; this deficiency reflects the requirement for erythrose-4-phosphate as a precursor for the synthesis of AAAs such as phenylalanine, tyrosine, and tryptophan (Josephson and Fraenkel 1969). *S. cerevisiae* harboring a deletion in the *TKL1* (one of two yeast TKL-encoding paralogs) was still able to grow in synthetic complete medium lacking AAAs (Sundström et al. 1993). This observation suggested that *S. cerevisiae* possesses two isogenes of *TKL* (*TKL1* and *TKL2*), and that *TKL2* could complement the AAAs synthesis function of *TKL1* (Schaaff-Gerstenschläger et al. 1993). However, the detailed mechanisms of regulation and roles of two paralogs remained unclear.

In the present study, we identified two *TKL* isogenes from *M. megachiliensis* (*MmTKL1* and *MmTKL2*). To investigate the role of these two genes in cell metabolism under conditions of stress, we analyzed (1) the structure of the genomic regions flanking the *MmTKL1* and *MmTKL2* coding regions and (2) the expression profiles and functions of each gene in relation to stress response and erythritol biosynthesis. This work provides new insights into the physiological significance of TKL isoforms and of erythritol biosynthesis in the cell protection systems of microorganisms. In addition, the results are expected to facilitate improved yields of erythritol, a promising biomaterial, via metabolic engineering.

## Materials and methods

### Strains and growth condition

The microbial strains and plasmids used in this study are listed in Table 1. *E. coli* DH5α (TOYOBO, Osaka, Japan), used as a host cell for molecular constructs, was cultured overnight in LB medium (2.0% tryptone, 1.0% yeast extract, 2.0% NaCl) at 37 °C. *M. megachiliensis* SN-124A (National Food Research Institute, Microbial Gene Bank, Tsukuba, Japan) was pre-cultured in 100 mL GY medium (2% glucose and 0.5% yeast extract) or YPD medium (1.0% yeast extract, 2.0% peptone, 2.0% glucose) in a 500-mL flask at 30 °C and 200 rpm until the OD_600_ reached 1.0. The pre-culture then was used as a 1% inoculum (i.e., 10 mL into 1 L; in a 3-L flask) in GY or YPD medium, and the resulting culture was further cultivated at 30 °C and 160 rpm until the OD_600_ reached 1.0.

**Table 1:** Fungal strains and plasmids used in this study

Strain or plasmid	Genotype or relevant features	Source
Strain		
*Moniliella megachiliensis*		
SN-124A	Wild	NFRI
*Saccharomyces cerevisiae*		
BY4741	MATa; *his3*Δ1; *leu2*Δ0; *met15*Δ0; *ura3*Δ0	EUROSCARF
BY4741Δ*tkl1*	MATa; *his3Δ*1; *leu2Δ*0; *met15Δ*0; *ura3Δ*0; *YPR074C*: *kanMX4*	EUROSCARF
Plasmid		
pDB05	pDEST32 containing a 70 bp *Hin*dIII and *Sac*I fragment from pBluescript II SK(+)	Yoshida et al. (2013)
p*MmTKL1*	pDB05 containing a *Not*I fragment of *MmTKL1* ORF	This study
p*MmTKL2*	pDB05 containing a *Not*I fragment of *MmTKL2* ORF	This study
p*ScTKL1*	pDB05 containing a *Not*I fragment of *ScTKL1* ORF	This study

For short-term stress loading, the 1-L GY or YPD cultures then were divided into 100-mL aliquots, and individual aliquots were supplemented by the addition of a stressing agent (glucose, NaCl, or menadione). Specifically, stressing agents were added to yield final concentrations as follows: 20% (w/v) glucose in GY medium, 1.2 M NaCl in YPD medium, or 0.15 mM menadione in YPD medium. The resulting 100-mL cultures then were incubated at 30 °C and 200 rpm for up to 120 min.

For long-term stress loading, pre-culture prepared as above was used as 1% inoculum in 100 mL of GY medium containing 20% glucose. The resulting culture was incubated at 30 °C and 200 rpm for 72 h, and aliquots were harvested for analysis every 12 h.

### Cloning of *TKL* genes from *M. megachiliensis*

The partial DNA sequence of a gene encoding a microbial *TKL* homolog was obtained from the cDNA library of *M. megachiliensis* SN G-42 (mutant strain of *M. megachiliensis* SN-124A). Primers specific for that sequences were designed and used to screen (via direct phage plaque PCR) a *M. megachiliensis* SN-124A genomic phage library for that *TKL*-encoding clone. The nucleotide sequence of the recovered clone was determined and used to design additional primers for recovering the full-length gene (designated *MmTKL1*) from the genomic phage library. Homology searches of the *M. megachiliensis* SN-124A draft genome sequence with *MmTKL1* identified a second gene encoding a *TKL* homolog. The corresponding DNA sequence was used to design gene-specific primers, which were in turn used to PCR amplify the second gene (designated *MmTKL2*) using *M. megachiliensis* SN-124A cDNA as the template. The nucleotide sequence of the recovered clones were determined and used to clone genomic sequences corresponding to the region upstream of *MmTKL2*. *MmTKL1* and *MmTKL2* then were separately cloned into the pGEM^®^ T-easy vector (Promega, Co., Madison, WI). The primers used in this experiment are listed in Table 2. Similarly, the amplified *TKL1* gene fragment was obtained from cDNA of *S. cerevisiae* BY4741 using PCR with degenerate primers, ScTKL1-Met-forward and ScTKL1-Stop-reverse (Table 2). The resulting fragment was cloned into pGEM^®^-T Easy vector (Promega Corporation, Madison, WI, USA).

**Table 2:** Primers used in this study

Primer name	Sequence (5′ → 3′)
Sequence analysis and cloning primers	
MmTKL1-forward1	GTCGAGTTGCCTTTGGTG
MmTKL1-reverse1	AGAACGAACCAAATCCAG
MmTKL1-met-*Not*I-forward	AAAGCGGCCGCATGCCACTCAAATCGTTTGA
MmTKL1-stop-*Not*I-reverse	AAAGCGGCCGCTTACGCCAACATTTCTTTGA
MmTKL2-met-forward1	ATGGTTGGGTTGGGTTGGGGCTGCCATGCT
MmTKL2-stop-reverse1	TTAAAGCAATTGTTTAACCTTCTTTTGAAT
MmTKL2-forward2	TACACTCCTGTTGGGCTC
MmTKL2-reverse2	CAGCTGCATCAACGACAC
T7	TAATACGACTCACTATAGGG
sp6	ATTTAGGTGACACTATAGAA
ScTKL1-met-forward	ATGACTCAATTCACTGACATTGATAAGCTA
ScTKL1-stop-reverse	TTAGAAAGCTTTTTTCAAAGGAGAAATTAG
Gene expression analysis primers	
GAPDH-RT-forward	CAAGGGTGGTGCCAAGAAGGT
GAPDH-RT-reverse	CCTTGGGGTCGTACGATTCG
MmTKL1-RT-forward	ACCTGGAAGGATCGACAGG
MmTKL1-RT-reverse	CGGTAAGTACCTTCGGGT
MmTKL2-RT-forward	CCTACTCGTCTGTACTTCCC
MmTKL2-RT-reverse	CCGAGTCAGATCCTTCCT

### Nucleotide sequence analysis

The nucleotide sequences of DNAs were analyzed using the BigDye^®^ Terminator v3.1/1.1 Cycle Sequencing Kit (Applied Biosystems, Tokyo, Japan) with an ABI automatic sequencer (PerkinElmer Japan, Tokyo, Japan). The nucleotide and amino acid sequence data were processed using GENETYX-Mac NETWORK software, version 15 (GENETYX CORPORATION, Tokyo, Japan). For both *MmTKL1* and *MmTKL2*, the 1000 bp upstream of each gene were searched using the TFSEARCH database for potential transcription factor binding motifs.

### Yeast transformation

A yeast centromere plasmid vector was constructed to evaluate whether *MmTKL1* and *MmTKL2* can complement the *S. cerevisiae* BY4741 *Δtkl1* mutant and rescue the phenotypes associated with this mutation, the growth deficiency in the SC plate lacking AAAs. Specifically, the multi-cloning site of pBluescript II SK (+) was inserted into a yeast centromere plasmid vector (pDEST32; Invitrogen, Carlsbad, CA, USA) that had been digested with *Hin*dIII and *Sac*I; the resulting episome was designated pDB05 (Yoshida et al. 2013). *Not*I-ended fragments harboring *MmTKL1*, *MmTKL2*, or *ScTKL1* (obtained from the corresponding clones in the pGEM^®^ T-easy vector) were ligated into *Not*I-digested pDB05 to generate pDB05-*MmTKL1*, pDB05-*MmTKL2*, or pDB05-*ScTKL1* respectively. Plasmids with and without inserts were separately transformed into the *S. cerevisiae Δtkl1* mutant using the lithium chloride method (Finlayson et al. 1991).

### Growth test of transformants

Leu^+^ transformants were selected on SC medium (synthetic complete medium) lacking leucine (SC-Leu). To evaluate the function of the *MmTKL* genes in vivo, the transformants were grown to logarithmic growth phase in SC medium. Following the cell pellets were resuspended in sterile distilled water at densities of 2 × 10^7^ cells/mL. Suspensions were subjected to six steps of tenfold serial dilutions, and the dilutions were spotted (5 µL/spot) on parallel plates of SC agar medium with and without added AAAs (tryptophan, phenylalanine, and tyrosine).

### Total RNA preparation and cDNA synthesis

Total RNA was extracted from *M. megachiliensis* using the previously described lithium chloride method (Iwata et al. 2015). An aliquot of 50 µg of total RNA then was treated with recombinant DNase I (Takara Bio, Shiga, Japan), extracted with PCI (phenol: chloroform: isoamylalcohol = 25:24:1), and purified by ethanol precipitation. cDNA was synthesized from 5 µg of the resulting RNA preparation using PrimeScript™ Reverse Transcriptase (Takara Bio, Shiga, Japan) according to the manufacturer’s protocol.

### Semi-quantitative PCR and real-time PCR

Semi-quantitative PCR amplification was performed using Go Taq^®^ polymerase (Promega, Madison, WI). The reaction mixtures were prepared using the manufacturer’s protocol. One round of PCR consisted of denaturation at 95 °C for 30 s, annealing at 58 °C for 30 s, and extension at 72 °C for 30 s. The amplified DNA fragments were detected by agarose-gel electrophoresis. Real-time quantitative PCR was performed using reaction mixtures incorporating Thunderbird SYBR qPCR Mix (TOYOBO, Osaka, Japan) according to the previously reported method (Iwata et al. 2015). The amplification of each cDNA template was performed in triplicate independently using a CFX96 real-time PCR system (Bio-Rad Laboratories, Inc., CA, USA). The primer sets used for PCR amplification are listed in Table 2.

### Determination of intracellular polyol content

The amount of intracellular polyol was determined as previously described (Kobayashi et al. 2013). Briefly, cell suspensions of *M. megachiliensis* were immersed in boiling water for 10 min, mixed with 100% (w/v) trichloroacetic acid, and vigorously stirred with a vortex mixer for 20 min at room temperature. After centrifugation at 13,400×*g* for 10 min, polyol in the supernatant was amperometrically assayed using a 4 mm × 250 mm CarboPac MA-1 column with an ICS-3000 chromatographic system (Dionex, Osaka, Japan) at flow rate of 0.3 mL of 500 mM NaOH as solvent per minutes. For dry cell weight determinations, cell suspensions (1 mL each) were dried by incubating for 5 h in a drying oven maintained at 80 °C. The polyol and dry cell weight examination were performed in triplicate independently.

## Results

### Cloning and sequencing of *MmTKL* genes from *M. megachiliensis*

Two transketolase isogenes were isolated from *M. megachiliensis*; these genes, designated *MmTKL1* (Accession Number LC163538) and *MmTKL2* (Accession Number LC163539), were identified from the genomic phage library and draft genome sequence data, respectively. *MmTKL1* consists of a 1956-bp ORF predicted to encode a 652-amino-acid protein with an estimated molecular mass of 70.8 kDa. This amino acid sequence shows 61% identity to putative or confirmed transketolases from *Cryptococcus bacillisporus* WM276 (Accession Number ADV_20663.1)*, C. neoformans* var. neoformans JEC21 (Accession Number XP_570357. 1), *Puccinia granminis* f. sp. *tritici* CRL 75-36-700-3(Accession Number EFP74700. 1), and *Coprinopsis cinerea* okayama7#130 (Accession Number XP_001836588. 2), and 28% identity to the *S. cerevisiae* transketolase 1 (Accession Number NP_015399). *MmTKL2* also consists of a 1956-bp ORF encoding a 652-amino-acid protein with an estimated molecular mass of 70.8 kDa. This amino acid sequence exhibits 66% identity to the *Cryptococcus gatti* WM276 transketolase (Accession Number XP_003192450), 65% identity to the *C. neoformans* var. *neoformans* JFC21 transketolase (Accession Number XP_570357.1), and 26% identity to the *S. cerevisiae* transketolase 1 (Accession Number NP_015399). MmTKL1 and MmTKL2 show 74% identity with each other.

**Fig. 1:**
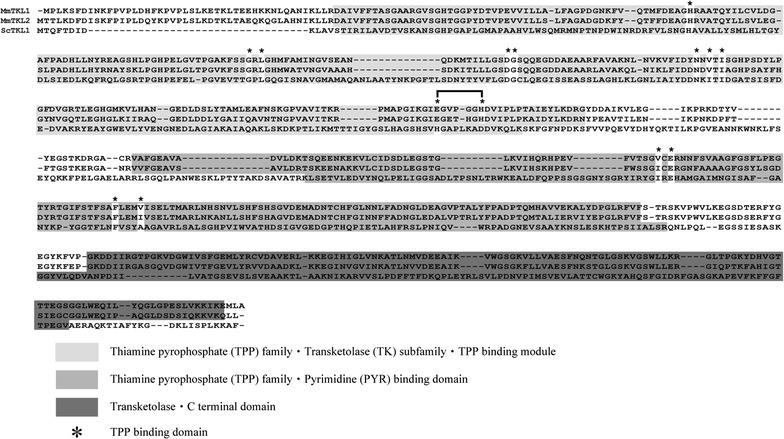
Alignment of amino acid sequence and domain structure of transketolases. MmTKL1, MmTKL2, and ScTKL1 indicate respectively the transketolase 1 and transketolase 2 proteins from *Moniliella megachiliensis* and the transketolase 1 from *Saccharomyces cerevisiae*. *Light gray zone* thiamine pyrophosphate (TPP) family, transketolase subfamily, TPP-binding module; *gray zone* thiamine pyrophosphate (TPP) family, pyrimidine (PYR)-binding module; *dark gray zone* transketolase, C-terminal module

BLAST search analysis revealed that both MmTKL1 and MmTKL2 are composed of domains typically shared among known TKLs (Fig. 1). From the N- to C-termini, these domains correspond to the TPP-binding module of the TKL subfamily of the thiamine pyrophosphate (TPP) family; the pyrimidine (PYR) -binding domain of 1-deoxy-D-xylulose-5-phosphate synthase (DXS); and the C-terminal domain of TKLs. The Ile189, Ile416, and Ala449 residues present in the TPP-binding site of *S. cerevisiae* TKL1 appear to correspond to respectively Val205, Val377, and Val413 in MmTKL1, and to respectively Val206, Ile414, and Ala449 in MmTKL2.

**Fig. 2:**
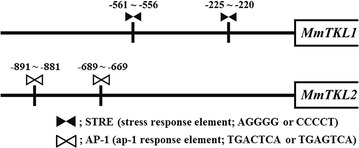
Putative stress response element (STREs) and Ap-1 response elements (AP-1 s) detected upstream of the *MmTKL1* and *MmTKL2* ORFs. Analysis was limited to the 1000 bp upstream of each ORF. *Black bow tie* STRE, *gray bow tie* AP-1 element, numerals represent base position with respect to ORF start codon

Two putative STREs (stress response element: AGGGG or CCCCT; binding sites involved in osmotic stress response) were detected in the sequences upstream of the *MmTKL1* ORF (at −556 to −561 bp and −220 to −225 bp with respect to the start codon).In contrast, two putative AP-1 s (activator protein 1 response elements: TGACTCA or TGAGTCA; binding sites involved in oxidative stress response) were detected in the sequences upstream of the *MmTKL2* ORF (at −891 to −881 bp and −689 to −669 bp with respect to the start codon) (Fig. 2).

### In vivo complementation by MmTKL1 and MmTKL2

**Fig. 3:**
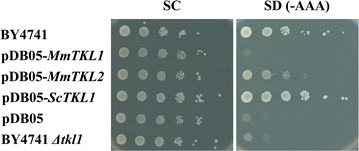
Complementation of *Saccharomyces cerevisiae tkl1* mutant by *MmTKL1* and *MmTKL2.* This assay tested complementation of the aromatic amino acid (AAA) auxotrophy of the *S. cerevisiae* BY4741 Δ*tkl1* mutant by plasmids harboring *MmTKL1*, *MmTKL2*, or *ScTKL1* (the endogenous *S. cerevisiae* gene) or with no insert (pDB05). Also tested were the untransformed mutant and the wild-type parent (BY4741). Growth was tested by *spotting* serial dilutions at an estimated 10^5^–10^0^ cells/spot, *left* to *right* on parallel synthetic complete (SC) agar medium with or without added AAAs (tryptophan, tyrosine, phenylalanine) and incubating the plates at 30 °C for 3 days

To investigate whether *MmTKL1* and *MmTKL2* can function in vivo as TKLs, we evaluated the ability of each gene to complement a *S. cerevisiae tkl1* mutant (An equivalent mutant of *M. megachiliensis* has not yet been constructed). Complementation was evaluated based on cell growth on SC agar medium lacking AAAs (SC-AAA), since TKLs are involved in AAA biosynthesis via effects on the supply of erythrose-4-phosphate. Both the parent strain (*S. cerevisiae* BY4741) and a complemented mutant [*S. cerevisiae tkl1* transformed with a plasmid containing the *S. cerevisiae* gene (*ScTKL1*)] grew well on SC-AAA plates. The yeast mutant transformed with the *MmTKL2* plasmid showed significant growth on the SC-AAA plate, although this growth was less vigorous than that observed in the positive controls. In contrast, growth of the yeast mutant harboring the *MmTKL1* plasmid was indistinguishable from that of the uncomplemented mutant or from the mutant transformed with empty plasmid (pDB05) (Fig. 3).

### Expression of *MmTKL* genes and erythritol production under short-term osmotic stress

**Fig. 4:**
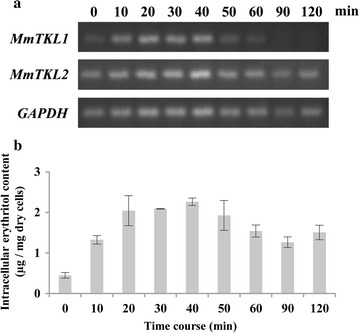
Gene expression and intracellular erythritol accumulation under short-term osmotic stress with 20% glucose in *Moniliella megachiliensis*. **a** Levels of gene expression were analyzed by semi-quantitative PCR analysis; data provided are representative ultraviolet-illuminated bands following electrophoresis in ethidium bromide-stained agarose gels. **b** Intracellular erythritol content is presented as µg/mg dry cells (mean ± SD from three independent determinations). When OD_600_ reached approximately 1.0, glucose was added to the cell culture to a final concentration of 20%, and the culture was incubated for 120 min at 30 °C. Gene expression was analyzed by semi-quantitative PCR using *GAPDH* as a standard. Intracellular erythritol content was chromatographically determined with an ICS-3000 system, and is represented as µg/mg dry cells

**Fig. 5:**
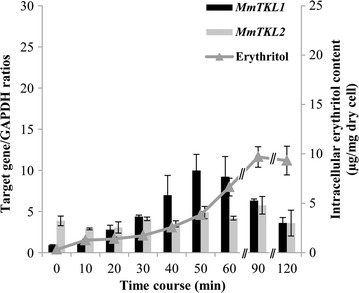
Gene expression and intracellular erythritol accumulation under short-term osmotic stress with 1.2 M NaCl in *Moniliella megachiliensis*. When OD_600_ reached approximately 1.0, NaCl was added to the cell culture to a final concentration of 1.2 M, and the culture was incubated for 120 min at 30 °C. Gene expression was analyzed by real-time PCR, and normalized using *GAPDH* expression level as a standard. Intracellular erythritol content was determined chromatographically with an ICS-3000 system, and is presented as µg/mg dry cells

Gene expression in *M. megachiliensis* was analyzed using semi-quantitative PCR during short-term (120-min) growth under various hyperosmotic conditions. In the presence of 20% glucose, the expression level of *MmTKL2* did not change with time, while that of *MmTKL1* gradually increased up to 40 min before subsequently falling (Fig. 4a). In the presence of 1.2 M NaCl, *MmTKL1* transcript accumulated with time, reaching a peak at 50 min 11-fold higher than baseline at 0 min (Fig. 5). However, hypersalinity did not result in apparent changes in *MmTKL2* expression levels through 120 min.

In short-term osmotic stress in 20% glucose medium, intracellular erythritol accumulation appeared to track with that of the *MmTKL1* transcript, with erythritol levels peaking at fivefold above baseline at 40 min before subsequently falling gradually (Fig. 4b).

### Expression of *MmTKL* genes and erythritol production under short-term oxidative stress

**Fig. 6:**
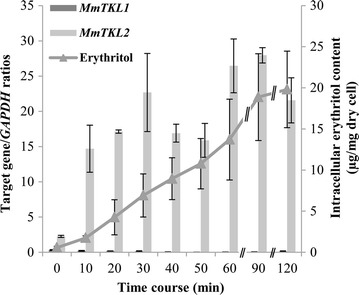
Gene expression and intracellular erythritol accumulation under short-term oxidative stress with 0.15 mM menadione in *Moniliella megachiliensis*. When OD_600_ reached approximately 1.0, menodione was added to the cell culture to a final concentration of 0.15 mM, and the culture was incubated for 120 min at 30 °C. Gene expression was analyzed by real-time PCR, and normalized using *GAPDH* expression level as a standard. Intracellular erythritol content was determined chromatographically with an ICS-3000 system, and is presented as µg/mg dry cells

Under oxidative stress (induced by supplementation with 0.15 mM menadione), the expression profiles of *MmTKL1* and *MmTKL2* differed considerably. *MmTKL2* showed marked induction of expression during the 120-min time course, with transcript levels after 90 min peaking at 12-fold over those at baseline (0 min) (Fig. 6). In contrast, levels of *MmTKL1* remained low throughout the 120 min of oxidative stress (Fig. 6). Notably, the patterns of expression of the two genes under oxidative stress were complementary to the patterns seen under osmotic stress with glucose or NaCl. Intracellular erythritol levels under oxidative stress increased with time through 120 min.

### Expression of *MmTKL* genes and erythritol production during long-term growth

**Fig. 7:**
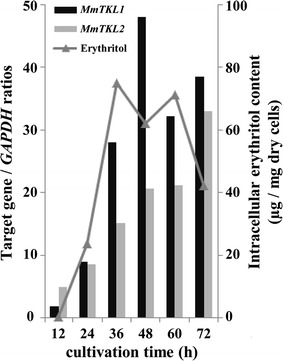
Gene expression and intracellular erythritol accumulation during long-term growth. *M. megachiliensis* was cultivated at 30 °C for 72 h in GY medium containing 20% glucose. Every 12 h, samples were removed for analysis. Gene expression was analyzed by real-time PCR, and normalized using *GAPDH* expression level. Intracellular erythritol content was determined chromatographically with an ICS-3000 system, and is presented as µg/mg dry cells

Gene expression analysis also was performed during long-term (72-h) growth under various stress conditions. In medium containing 20% glucose, expression of both *MmTKL1* and *MmTKL2* began increasing rapidly starting at around 12 h (early exponential growth) (Fig. 7). The levels of *MmTKL1* transcript peaked at 48 h before subsequently decreasing slightly; in contrast, *MmTKL2* transcript continued to accumulate through 72 h under these conditions.

In long-term osmotic stress in 20% glucose medium, intracellular erythritol levels increased rapidly starting at around 24–36 h after inoculation, with the increase tracking with those of both *MmTKL1* and *MmTKL2* expression. Peak erythritol levels were 40-fold higher than those at 12 h (Fig. 7).

## Discussion

Two *TKL*-encoding isogenes (*MmTKL1* and *MmTKL2*) were cloned and sequenced from *M. megachiliensis*, a hyper-osmotolerant basidiomycetous yeast-like fungus. The amino acid sequences of the MmTKL1 and MmTKL2 proteins exhibited lower levels of identity to that of *S. cerevisiae* TKL1 (28 and 26%, respectively) than to those of other basidiomycetous fungi. Strikingly, several amino acid residues known to be involved in TPP binding were substituted in the MmTKL proteins compared to the corresponding residues of ScTKL1. However, all these substitutions were conservative, representing amino acids of the same family with hydrophobic side chains. Thus, despite sequence divergence, MmTKL1 and MmTKL2 were expected to possess functions similar to those of the TKLs of *S. cerevisiae*.

The MmTKL1 and MmTKL2 proteins were found to exhibit strongest homologies (61–66% identity) to TKL proteins of *Cryptococcus* or *Puccinia* species. Pathogenic *Cryptococcus* (Wong et al. 1990) and *Aspergillus* (Wong et al. 1989) have been reported to accumulate high levels of mannitol in response to hyper-osmotic environments, a strategy that enhances survival when infecting host cells. *S. cerevisiae* or *Candida glycerinogenes* also are known to accumulate glycerol in response to conditions of hyper-osmosis, with glycerol serving as an osmo-regulatory-compatible solute (O’ Rourke et al. 2002; Chen et al. 2008). Under hyper-osmotic conditions, the *S. cerevisiae* Hog1 protein (the downstream-most protein kinase of the HOG (high osmolarity glycerol) pathway is activated via phosphorylation and rapidly translocates to the nucleus (Edmunds and Mahadevan 2004). Upon translocation to the nucleus, phosphorylated Hog1 (in cooperation with other transcription factors) stimulates transcription of the genes encoding *GPD1* (glycerol-3-phosphate dehydrogenase 1) and *GPP2* (glycerol-3 phosphate phosphatase 2) via STREs located upstream of the corresponding genes, resulting in glycerol biosynthesis (Alepuz et al. 2001; Ansell et al. 1997). Hence, STREs are believed to play an essential role in the osmotic stress response in yeast cells. We previously reported that *M. megachiliensis* possesses three erythrose reductase isogenes (*ER1*, *ER2*, *ER3*) and two transaldolase isogenes (*TAL1*, *TAL2*); the products of these genes are known to be involved in erythritol biosynthesis (Kobayashi et al. 2013, Iwata et al. 2015). Among these genes, *ER3* and *TAL2* harbor putative STREs within 1000 bp upstream of the respective ORFs. In the present work, we noted that the *MmTKL1* ORF is preceded (at −556 and −220 bp) by two upstream putative STREs; in contrast, the *MmTKL2* ORF appears to lack STREs within 1000 bp upstream of the initiation codon. Hence, we postulate that *MmTKL1* is involved in regulation of the osmotic-stress response via the PPP. On the other hand, two putative AP-1 elements were found upstream of the *MmTKL2* ORF, but not proximal to *MmTKL1*. Since AP-1 has been shown in other organisms to mediate responses to oxidative stress (Toone and Jones 1999), we hypothesize that *MmTKL2* is involved in the oxidative stress response. Similar results have been obtained for *MmTAL1* (Iwata et al. 2015).

In addition to stress response, the function of *MmTKL1* and *MmTKL2* was evaluated based on another criterion: nutritional requirement. The erythrose-4-phosphate generated by TKLs can be converted (via the PPP; in microorganisms, fungi, and plants) to AAAs by way of the shikimic acid pathway (Hermann and Weaver 1999). Notably, a *S. cerevisiae tkl1* deletion mutant is not able to grow in synthetic complete medium lacking AAAs. Our transformation tests demonstrated that *MmTKL2* (but not *MmTKL1*) can partially complement the AAA auxotrophy of a *S. cerevisiae tkl1* mutant. We have confirmed this result, including demonstration that the *MmTKL1* plasmid is indeed present in the *S. cerevisiae* transformant (data not shown). The reason for this failure to complement is unknown, but may reflect the absence of heterologous expression of *MmTKL1* in the yeast background under the plate assay conditions. Meanwhile, we are not convinced that *MmTKL1* and *MmTKL2* are orthlogs of *TKL1* and *TKL2* of *S. cerevisiae*, respectively, because number of *MmTKL* is not strictly defined. In fact, we have found three TKL homologues in *M. megachiliensis* draft genome sequence decoded, and obtained two of them, MmTKL1 and MmTKL2, as shown in this study. It is unclear that putative third *TKL* gene may complement *S. cerevisiae* TKL function. Analyses to determine *MmTKL1* expression in the transformant and further, putative third gene will be needed.

In the presence of 20% glucose in *M. megachiliensis*, endogenous *MmTKL1* expression peaked at 40 min after osmotic stress loading before subsequently gradually decreasing through 120 min. In contrast, *MmTKL2* showed an approximately constant expression level during this osmotic stress interval. Similar results were obtained for gene expression profiles under NaCl-induced osmotic stress. In contrast, distinct results were obtained under conditions of short-term (120-min) oxidative stress, with the level of *MmTKL1* expression remaining relatively low while *MmTKL2* transcript exhibited marked accumulation. These results implicate *MmTKL1* as a major mediator of the response to hyper-osmotic stress; in contrast, *MmTKL2* is inferred to be a major mediator in the response to oxidative stress. The oxidative stressor used here (menadione) is metabolized to semiquinone by the oxido-reductase system of the cell, and then subsequently converted to a quinone that generates reactive oxygen species (ROS) (Yamashoji et al. 1991). ROS often induce oxidative damage and impair cell survival (Yashiki and Yamashoji 1996). *S. cerevisiae TKL1* reportedly is induced by oxidative agents like hydrogen peroxide or acetoaldehyde (Jamieson 1998). Furthermore, TKL mediated by Yap1p and Skn7p in *S. cerevisiae* has been reported to contribute to the regulation of glutathione and NADPH for cell redox homeostasis (Carter et al. 2005; Slekar et al. 1996). We postulate that the ROS generated by menadione degradation similarly induces *MmTKL2* expression in *M. megachiliensis*, such that this isoform of TKL contribute to the regulation of glutathione and NADPH for eliminating ROS in this yeast-like fungus.

We used 72-h growth in medium containing 20% glucose to model long-term exposure to hyper-osmotic conditions; these conditions parallel those used in industrial fermentation for production of erythritol. Under these conditions, *MmTKL1* expression rapidly increased starting at 12 h and reached a maximum at 48 h, while *MmTKL2* expression increased throughout the 72-h experiment. A clear correlation was observed between *MmTKL1* expression and erythritol accumulation under conditions of hyper-osmotic glucose stress; no such correlation was observed between *MmTKL2* expression and erythritol production. As postulated for short-term stress, *MmTKL1* expression appears to be associated with the long-term response to osmotic stress in this organism. The expression of *MmTKL2* increased with time during long-term osmotic stress, and this isogene may contribute to elimination of ROS that accumulate during long-term stress in high-glucose culture, which is more or less similar to oxidative stress caused by menadione. Regarding stress responses, compensatory behavior of isogenes is known to apply to *S. cerevisiae GPD1* and *GPD2*, which encode isoforms of a key enzyme of glycerol biosynthesis (Ansell et al. 1997).

Based on the results obtained here, we consider that *MmTKL1* is involved in the *M. megachiliensis* response to osmotic stress. In contrast, *MmTKL2* appears to be involved in the response to oxidative stress, while also contributing to the AAA supply that is essential for growth in synthetic and minimal media. Intriguingly, *S. cerevisiae* also has been reported to possess two *TKL* isogenes, *TKL1* and *TKL2*. Based on mutant phenotype, *TKL1* is presumed to contribute to the supply of AAAs; the function of the *ScTKL2* isogene presumed complementary of TKL1 remains unclear.

In summary, our results suggest that *MmTKL1* and *MmTKL2* may play distinct and complementary roles in *M. megachiliensis* defense against environmental stress, mediated by induction of erythritol production. To our knowledge, the results obtained in our study are the first instance of complementary function of *TKL* isogenes in association with stress response. We are now going to analyze the detailed mechanism of erythritol biosynthesis involved in ROS elimination in stress response of *M. megachiliensis*.
